# Utility of magnetic resonance imaging in the diagnosis of unsuspected cases of Parsonage-Turner syndrome: two case reports

**DOI:** 10.1186/1752-1947-7-255

**Published:** 2013-11-07

**Authors:** Ishan Kumar, Ashish Verma, Arvind Srivastava, Ram C Shukla

**Affiliations:** 1 Department of Radiodiagnosis and Imaging, Institute of Medical Sciences, Banaras Hindu University, Varanasi 221005, Uttar Pradesh, India

## Abstract

**Introduction:**

MRI is becoming increasingly important in the evaluation of shoulder pain, especially in the diagnosis of rotator cuff injuries and conditions that mimic them. Parsonage-Turner syndrome is a well-defined clinical entity that presents with acute-onset shoulder pain and weakness, often first recognized on magnetic resonance imaging scans.

**Case presentation:**

We studied magnetic resonance imaging features of two Asian men (ages 24 and 31 years) who presented with variable-onset shoulder pain and weakness. Magnetic resonance imaging revealed increased T2-weighted signal intensity of supraspinatus and infraspinatus muscles in both patients.

**Conclusion:**

Magnetic resonance imaging findings are distinctive, although nonspecific, in cases of Parsonage-Turner syndrome, and knowledge of the imaging and clinical features of this disease enable clinicians to arrive at the correct diagnosis and guide appropriate management.

## Introduction

Pain in the shoulder joint is one of the most common causes of musculoskeletal consultation in modern orthopedic practice and is commonly attributed to rotator cuff and labral tears, which are efficiently managed surgically. The imaging of shoulder joints usually begins with plain radiographs; however, in most shoulder joint pathologies, it cannot be relied upon, and the modality which clearly stands out in shoulder imaging is magnetic resonance imaging (MRI). With the use of MRI, we are able to diagnose other conditions that mimic rotator cuff injury but can be managed conservatively, thus avoiding unnecessary surgery. One of these conditions is Parsonage-Turner syndrome (PTS), also known as idiopathic brachial neuritis, neuralgic amyotrophy or neuritis of the shoulder girdle. This syndrome, although relatively uncommon, often precipitates debilitating pain and weakness in the joint, and the apprehension it arouses in the patient can even lead to inappropriate surgery if overlooked. In this report, we share our experience with the cases of two patients with PTS who had different clinical backgrounds in whom MRI was performed. MRI revealed typical imaging features and hence had a positive impact on therapy.

## Case presentations

### Patient 1

**Figure 1:**
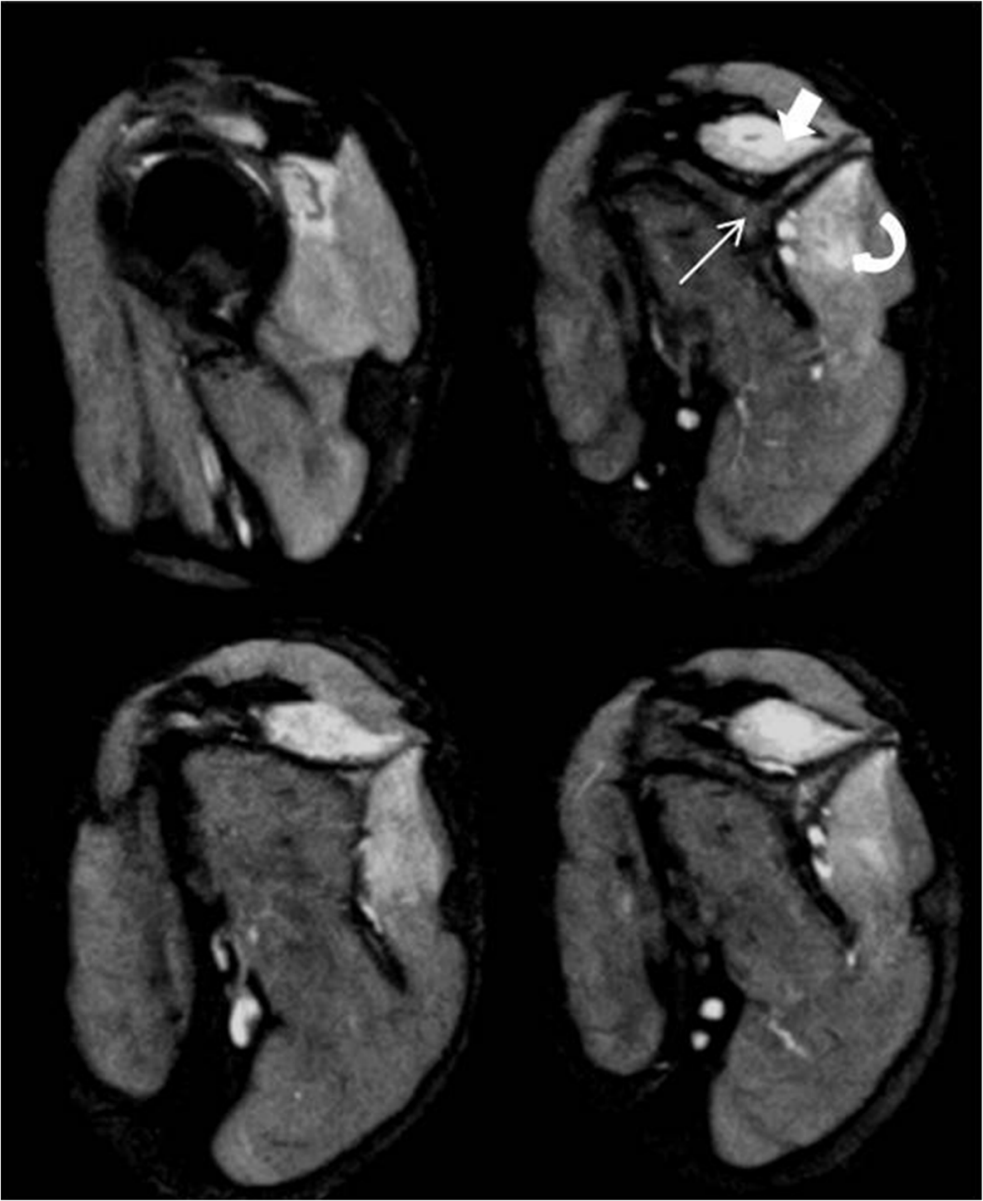
**Magnetic resonance imaging of patient 1 at the level of shoulder girdle including the periscapular muscles.** Sagittal T2-weighted fat-suppressed contiguous section at the level of shoulder showing edema in suprasinatus (solid arrow) and infraspinatus (curved arrow) muscles, respectively above and below the spine of scapula (straight arrow). The brachial plexus in this patient, however did not reveal any abnormality.

A 24-year-old Asian man presented to our institution with the complaint of an episode of gradually progressive right-sided neck pain that radiated into the right shoulder without associated numbness or tingling. The onset of the pain had occurred two days earlier. The patient attributed this to an abnormal posture in which he had slept the previous night. His physical examination revealed normal sensation and reflexes of both upper extremities. Decreased power was noted in the left upper limb. Cervical spine radiographs and MRI did not reveal any abnormalities. The patient’s presumption regarding the cause of his pain was taken as such, and he was given a combination of anti-inflammatory and anti-spasmodic drugs for symptomatic relief. The weakness in his left shoulder girdle muscles increased and he underwent MRI of the left shoulder approximately one week after the onset of symptoms. MRI of the left shoulder revealed diffusely bright signal intensity in the supraspinatus and infraspinatus muscles on T2-weighted images (Figure 1). This signal was not associated with any sign of rotator cuff injury or any changes in the brachial plexus. A diagnosis of acute brachial plexus neuritis was made. An electromyogram and nerve conduction studies were done subsequently, the results of which were consistent with the diagnosis of brachial plexus neuritis with severe subacute denervation in the supraspinatus and infraspinatus muscles. Distal median and ulnar motor and sensory and radial sensory nerve conduction studies were within normal limits, as were proximal median and ulnar motor nerve conduction studies.

The patient refused the advised treatment regimen, however, because of his reservations about taking steroids. He preferred physical therapy instead. After three weeks, his weakness worsened with further deceased power. The patient gradually improved after that, however, and at his last follow-up examination at four months, only mild difficulty with shoulder abduction existed.

### Patient 2

**Figure 2:**
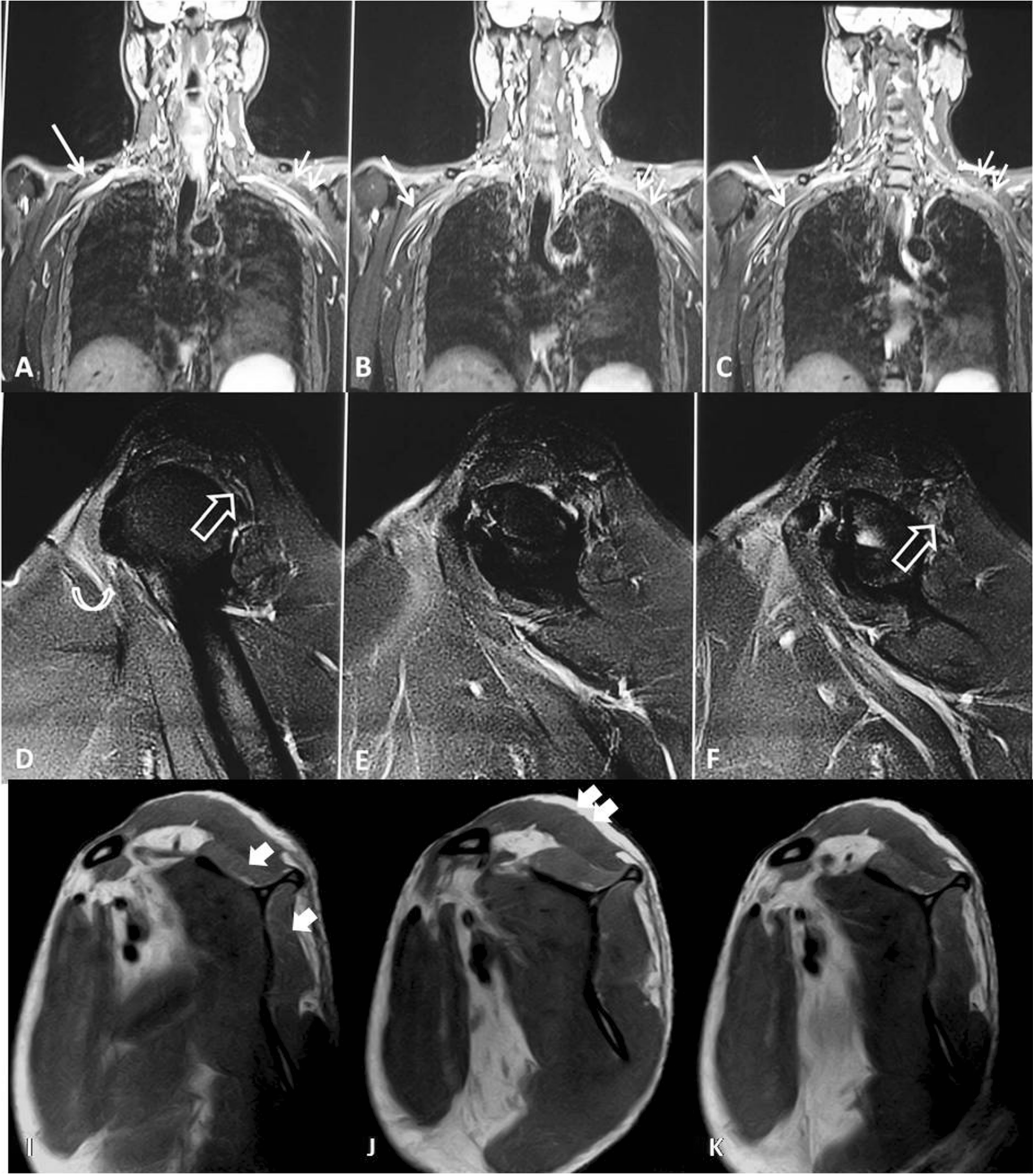
**Magnetic resonance imaging of patient 2 depicting brachial plexus as well as periscapular muscles. (A,B,C)** Coronal T2W fat suppressed contiguous section for brachial plexus shows edema in the trunks and cords of the right brachial plexus (straight arrow). Compare this to the normal brachial plexus on the left side (double arrow). **(D,E,F)** Sagittal T2-weighted fat-suppressed contiguous section at the level of the shoulder show edema and atrophy in the infraspinatus muscle (hollow arrow). Edema is also noted in the anterior fibres of the subscapularis muscle (curved arrow). **(I,J,K)** Comparable T1W sagittal images show fatty infiltration in the supraspinatus and infraspinatus muscles (solid arrows in I). Compare the increased intensity of these muscles as compared to the deltoid (double solid arrow in J), due to fatty infiltration.

A 31-year-old, right-handed Asian man presented to our institution with weakness and on-and-off burning pain in his right shoulder for the previous 3 months. The pain had gradually worsened over that time, and it interfered with his sleep on a few occasions. He denied any history of sensory disturbances. The patient provided a history of playing cricket since childhood, but he had been off the field for the past five years. His physical examination revealed normal sensation and reflexes of both upper limbs. Decreased power was noted in his right upper-limb muscles. Cervical spine radiographs showed features of early degenerative changes. Electromyographic and nerve conduction studies exhibited severe spontaneous fibrillation, positive waves and increased insertional activity in his supraspinatus and infraspinatus muscles, suggesting neurologic injury. MRI showed bright signal intensity in the supraspinatus and infraspinatus muscles on T2-weighted images without any changes in other muscles (Figure 2). Edema was noted in the right brachial plexus as well, without any evidence of brachial plexus trauma. A diagnosis of PTS was made. The patient was then started on oral prednisolone (1mg/kg/day) for two weeks, which was gradually tapered for another two weeks. His pain was relieved promptly, and his muscle weakness improved gradually. The patient was serially followed up for six months, during which time his muscle power improved to grade 4/5.

## Discussion

Acute brachial neuritis was described as early as 1897 [1], and it was further elaborated in the early 20th century in a study of 46 soldiers with shoulder pain [2,3]. Parsonage and Turner systematically reported varying disease patterns in their series of 136 patients and described this entity as neuralgic amyotrophy and shoulder girdle syndrome [3,4]. The cause of this disease remains controversial, but various hypotheses have been proposed, including post–viral immunological insult to the brachial plexus, immunization, pregnancy, surgery, radiation, heroin abuse or treatment with interferon [5,6]. A hereditary form of this disease, known as *hereditary neuralgic amyotrophy*, also has been described, which is linked to chromosome 17q24 [5]. In our patients, no predisposing factor could be found and no causal association could be inferred. An incidence of 1.64/100,000 person-years has been reported [7-10], with most cases occurring in the third to seventh decades of life [8]. A clear male predisposition has been reported, with a male-to-female ratio reported to range from 2:1 to 11.5:1 [8]. Both of the patients in our present report were men, one in his early fourth decade and the other in his third decade of life, respectively, and they presented to our institution over the course of four years. Both the patients in our study were right-handed with involvement of the dominant hand in one and the non-dominant hand in the other. No correlation could be inferred between handedness and involvement in the present report, and no correlation has been described to date in the literature [6,8,9]. Bilateral involvement has been reported in up to one-third of cases in strikingly asymmetrical fashion [9]. The disease typically starts suddenly, with severe pain followed by flaccid paralysis of the shoulder muscles when the pain abates. Usually, atrophy of one or more of the involved muscles develops, and the weakness commonly resolves spontaneously over a period of 6 to 18 months [5]. This classical chronology was followed in only one of our two patients (patient 1); the other patient (patient 2) presented with insidious onset pain during the course of two to three months with progressive muscle weakness over this period. Atypical presentation complicates the diagnosis. There have been reports of cranial nerve and phrenic nerve involvement. These cases have been misunderstood as diaphragmatic rupture or palsy, and often patients underwent extensive investigations to procure evidence of such misdiagnosis [3]. PTS most commonly involves suprascapular, axillary and long thoracic nerves individually or in combination [6]. In our study, both the cases revealed isolated suprascapular nerve involvement, and the supraspinatus and infraspinatus muscles were affected in both cases. These muscles have almost invariably been affected in most studies [9]. MRI findings in PTS usually reflect acute denervation edema or chronic atrophy with fatty infiltration. MRI remains the single most useful modality for diagnosis, with most cases being first suspected when a patient is referred for shoulder MRI after a likely sports injury. Acute changes are depicted best by bright signal intensity on T2 sequences with fat saturation within two weeks after denervation and may concur without any T1-weighted signal intensity change. These changes are thought to result from increased extracellular water content or increased capillary blood volume in the affected muscles. T1 sequences best depict chronic loss of muscle bulk and bright intramuscular intensity consistent with fatty infiltration [6,11]. Patient 1 in our report presented with acute-onset symptoms, and MRI revealed increased T2-weighted signal intensity without fatty atrophy on T1 sequences, which is consistent with acute denervation changes. Of note, however, were the MRI findings in patient 2, who presented with an insidious case history but showed acute T2-weighted signal hyperintensity without any evidence of fatty atrophy of muscles, as was expected on the basis of our current understanding of the pathophysiology of this disease. The treatment is conservative and based on physical therapy and treatment with analgesics and corticosteroids [5].

## Conclusion

Although the MRI findings are non-specific for this disease, the correlation of clinical data and exact chronological history points toward the diagnosis. Furthermore, MRI helps to exclude other mimics with similar symptoms, such as rotator cuff injury, impingement syndrome and entrapment neuropathies. Because of increasing referrals for MRI of patients with shoulder pain, knowledge of the imaging and clinical features of this entity are important because the correct diagnosis has definitive impact on guiding therapy and more accurately preventing overtreatment and unnecessary surgery.

## Consent

Written informed consent was obtained from the patients for publication of this case report and any accompanying images. A copy of the written consent is available for review by the Editor-in-Chief of this journal.

## Abbreviations

PTS: Parsonage-Turner syndrome; MRI: Magnetic resonance imaging.

## Competing interests

The authors declare that they have no competing interests.

## Authors’ contributions

IK and AV analyzed and interpreted the patient data regarding the clinical details, electromyographic findings and MRI findings and were the major contributors to the writing of the manuscript. The MRI findings were also independently analyzed by AS and RCS. All authors read and approved the final manuscript.
